# The predictive ability of Controlling Nutritional Status score on in-hospital mortality in patients admitted to coronary care unit

**DOI:** 10.1590/1806-9282.20240958

**Published:** 2024-12-02

**Authors:** Serdar Gökhan Nurkoç, Şeyhmus Atan, Mehmet Koray Adalı, Mevlüt Demir, Yunus Emre Yavuz, Burak Açar, Meltem Altınsoy, İbrahim Halil Tanboğa, Fatih Kahraman

**Affiliations:** 1 Yozgat City Hospital, Cardiology Clinic – Yozgat, Turkey.; 2 Ankara University, Faculty of Medicine, Department of Cardiology – Ankara, Turkey.; 3 Pamukkale University, Faculty of Medicine, Department of Cardiology – Denizli, Turkey.; 4 Kütahya Health Sciences University, Evliya Çelebi Training and Research Hospital, Cardiology Clinic – Kütahya, Turkey.; 5 Siirt University, Siirt Training and Research Hospital, Department of Cardiology – Siirt, Turkey.; 6 Kocaeli University, Faculty of Medicine, Department of Cardiology – Kocaeli, Turkey.; 7 Ankara Etlik City Hospital, Cardiology Clinic – Ankara, Turkey.; 8 Hisar Intercontinental Hospital, Department of Cardiology – İstanbul, Turkey.

**Keywords:** Coronary care unit, In-hospital mortality, Nutrition

## Abstract

**OBJECTIVE:**

Controlling Nutritional Status score was previously described and has been used in predicting short- and long-term outcomes in different patient populations. The aim of this study was to test the relationship between Controlling Nutritional Status score and in-hospital mortality in coronary care unit patients (MORCOR-TURK population).

**METHODS:**

In this multicenter and national study, all patients with an available Controlling Nutritional Status score were included in the analysis. The Controlling Nutritional Status score was calculated according to previously described criteria. To be able to understand the significance of the Controlling Nutritional Status score, we constructed two models. Model 1 included age, heart failure, chronic kidney disease, hypertension, diabetes mellitus, and coronary artery disease history. Model 2 included the Controlling Nutritional Status score and Model 1. We then statistically compared the performances of the two models.

**RESULTS:**

A total of 1,018 patients with known Controlling Nutritional Status scores were included in the analysis. Demographic characteristics are shown. In Model 1, the -2 log-likelihood ratio was 395.995, Nagelkerke R2 was 0.133, and area under the curve was 0.739 (95%CI 0.67–0.81). In the second model to which the Controlling Nutritional Status score is added (Model 2), the -2 log-likelihood ratio was 373.743, Nagelkerke R2 was 0.191, and area under the curve was 0.787 (95%CI 0.72–0.85). The area under the curve value of Model 2 was statistically higher than Model 1 (DeLong p-value: 0.01). A statistically significant correlation was found between death and Controlling Nutritional Status score in Model 2 [OR 1.347 (1.193–1.521), p<0.001].

**CONCLUSIONS:**

Our study showed that the Controlling Nutritional Status score may be a significant predictor of in-hospital mortality in coronary care unit patients.

## INTRODUCTION

Malnutrition due to several diseases is independently associated with high mortality rates. Additionally, prolonged and recurrent hospitalization is a negative prognostic indicator associated with greater resource consumption. Therefore, nutritional support is very important for patients in the intensive care unit (ICU). Loss of body mass may hinder functional recovery after discharge and reduce the chance of survival^1,2^.

Controlling Nutritional Status (CONUT) is a frequently used objective measure for assessing nutritional status. While numerous methods exist for measuring malnutrition to evaluate the pathophysiology of various diseases, the CONUT score, which assesses albumin, total cholesterol (TC), and total lymphocyte values, stands out as one of the most crucial indices^3-5^. Malnutrition detected by the CONUT score has been associated with an unfavorable prognosis in conditions such as chronic heart failure (HF) and peripheral artery disease. A study stated that malnutrition assessed through the CONUT score is associated with adverse outcomes in patients with stable coronary artery disease (CAD) undergoing percutaneous coronary intervention (PCI)^6-9^. In addition, several studies were conducted on patients with chronic HF and chronic liver disease using the CONUT score^10^.

The patient population previously admitted to the coronary care unit (CCU) for any reason has not been evaluated. In this study, we wanted to test the relationship between CONUT score and in-hospital mortality in CCU patients (MORCOR-TURK population).

## METHODS

### Study design

The MORCOR-TURK trial was a comprehensive study conducted across all seven geographical regions of Turkey, involving 50 critical CCUs. Registered on clinicaltrials.gov under NCT05296694, it was a multicenter, prospective, cross-sectional, and non-interventional study. Over a 1-month period, patient characteristics and short-term outcomes were systematically documented. Ethical approval for the study was granted by the Afyonkarahisar University of Health Sciences Clinical Research Ethics Committee (Approval Number: 2011-KAEK-2, Date: 05.08.2022), and the study strictly adhered to the principles of good clinical practice and the Declaration of Helsinki. Before taking part in the study, all participants or their legally authorized representatives provided written informed consent.

### Study population

Baseline characteristics, including demographics, risk factors, past medical history, hemodynamic grade, and laboratory findings, were methodically gathered for patients admitted to the CCU from September 1 to 30, 2022 (Table 1). Exclusion criteria were dysphagia, active cancer, severe cognitive impairment, long-term stay in CCU for social reasons, patients without written consent, and being under 18 years of age.

**Table 1:** Demographic and clinical characteristics of the patient population.

	All patients (n=1,018)
Age (years)	67 (57–75)
Male gender, n (%)	663 (65.1)
Hypertension, n (%)	638 (62.7)
Diabetes mellitus, n (%)	376 (36.9)
Dyslipidemia, n (%)	330 (32.4)
Stroke*, n (%)	59 (5.8)
CAD, n (%)	471 (46.3)
AF, n (%)	191 (18.8)
Heart failure, n (%)	355 (34.9)
Chronic renal disease, n (%)	189 (18.36)
EF (%)	50 (40–55)
Heart rate (bpm)	80 (70–96)
Mean blood pressure (mmHg)	94 (83–105)
Glucose (mg/dL)	127 (103–173)
Glomerular filtration rate (mL/min)	73 (49–93)
Sodium (mEq/L)	138 (135–140)
Potassium (mEq/L)	4.3 (4–4.8)
Calcium (mEq/L)	9 (8.6–9.3)
Magnesium (mEq/L)	1.9 (1.7–2.1)
CRP (mg/L)	6.8 (2.1–22)
Albumin (g/dL)	3.9 (3.5–4.3)
Hematocrit (%)	39.4 (35–43.4)
WBC (103/µL)	9.3 (7.2–11.6)
Platelet count (103/µL)	227 (184–279)
Total cholesterol (mg/dL)	152 (125–175)
Triglyceride (mg/dL)	108 (77–148)
HDL (mg/dL)	38 (32–47)
LDL (mg/dL)	90 (67–110)

Continuous variables are presented as mean±SD or median (IQR), and categorical variables are presented as frequency (%). AF: atrial fibrillation; bpm: beat per minute; CAD: coronary artery disease; CRP: C-reactive protein; EF: ejection fraction; HDL: high-density lipoprotein; LDL: low-density lipoprotein; WBC: white blood cell.

*Includes both ischemic and hemorrhagic stroke cases.

Upon admission, standard biochemical tests, complete blood counts, cardiac biomarkers, and lipid profiles were collected and analyzed.

The CONUT score was calculated according to previously described criteria. The predictive value of the CONUT score was evaluated by logistic regression analysis. To be able to understand the significance of the CONUT score, we constructed two models. Model 1 included age, HF, chronic kidney disease, hypertension, diabetes mellitus, and CAD history. Model 2 included the CONUT score and Model 1.

### Statistical analysis

Statistical analyses were conducted using the Statistical Package for the Social Sciences software program version 23.0 (SPSS Inc., Chicago, IL, USA). Nonparametric variables were presented as median (interquartile range) and compared using either the Mann-Whitney U test or the Kruskal-Wallis test. Categorical variables were expressed as counts and percentages, and comparative analysis was performed using either the chi-square test or Fisher’s exact test. The duration of survival in the CCU was calculated from the date of admission to either death or discharge. A p<0.05 was considered statistically significant.

## RESULTS

**Figure 1:**
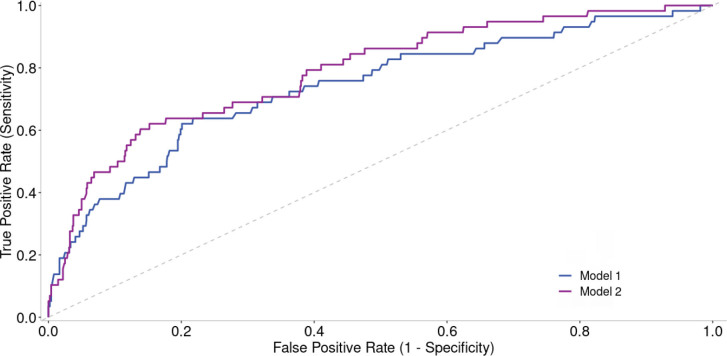
Comparison of Model 1 and Model 2 in predicting in-hospital mortality. Model 1: Age, heart failure, chronic kidney disease, hypertension, diabetes mellitus, coronary artery disease. Model 2: Model 1+Controlling Nutritional Status score.

A total of 1,018 patients with known CONUT scores were included in the analysis. The mean CONUT score was 2.74±1.9 for the whole population. The median age was 67 (range: 57–75) years, and patients were mostly male (n=663 [65.1%]). In the study group, 62.7% (n: 638) of the patients had hypertension, 36.9% (n: 376) had diabetes, and 46.3% (n: 471) had CAD. The number of patients with stroke in the patient population included in the study was relatively low, 5.8% (n: 59). Additionally, the average albumin value was calculated as 3.9 g/dL (3.5–4.3). Demographic characteristics are shown in Table 1. The in-hospital mortality rate of the whole population was 5.7% (58 patients), and the CONUT score was significantly higher in non-survivors [4 (2–6.3) vs 2 (1–3.8), p<0.001]. In univariate analysis of the CONUT score of the receiver operating characteristic (ROC) curve, the -2 log-likelihood ratio was 397.354, Nagelkerke R2 was 0.129, and AUC was 0.758 (95%CI 0.69–0.81). In Model 1, the -2 log-likelihood ratio was 395.995, Nagelkerke R2 was 0.133, and AUC was 0.739 (95%CI 0.67–0.81). In the second model to which the CONUT score is added (Model 2), the -2 log-likelihood ratio was 373.743, Nagelkerke R2 was 0.191, and AUC was 0.787 (95%CI 0.72–0.85). The area under the curve value of Model 2 was statistically higher than Model 1 (DeLong p-value: 0.01) (Figure 1). A statistically significant correlation was found between death and CONUT score in Model 2 [OR 1.347 (1.193–1.521), p<0.001].

## DISCUSSION

Patients in the CCU are primarily monitored for ACS, with a focus on coronary PCI. Nevertheless, accurate evaluation and treatment of malnutrition are crucial. However, due to practical challenges, nutritional assessments often go overlooked. Many patients, particularly the elderly and those admitted for ACS, may experience issues related to malnutrition. Furthermore, this condition can be exacerbated by trauma, sepsis, and other factors11,12. The incidence of both malnutrition and comorbid diseases rises with advancing age. Consequently, there is an increased mortality rate attributed to malnutrition in the elderly patient population13.

Studies on nutritional assessment tools and their ability to predict survival rates in intensive care patients have yielded mixed results. One study found that parameters such as serum albumin, serum pre-albumin, transferrin, retinol-binding protein, and lymphocyte values were inadequate for predicting outcomes in critically ill patients^14^. Recent studies have demonstrated that the CONUT score is associated with poor prognosis in various cardiovascular diseases. It has been noted that a high CONUT score increases the rates of hospitalization and mortality due to HF in patients with both acute and chronic conditions^15^. The CONUT score was identified as an independent predictor of adverse cardiovascular and extremity events in patients with peripheral artery disease. Additionally, in another study, high CONUT scores were linked to unfavorable long-term outcomes in patients with stable CAD undergoing PCI^7,8^. Furthermore, there are several studies indicating that the prognostic nutritional index (PNI) or CONUT is associated with the severity of chronic liver disease. A prospective study showed the correlation of increased cardiovascular events with higher CONUT and lower PNI scores in patients with chronic HF^6^. In another study, the CONUT score was developed as one of the most useful indices reflecting malnutrition status in the hospital population^3^. Rinninella et al. found the CONUT score to be a reliable and simple predictor of long-term hospital stays and in-hospital mortality^16^. Buglio et al. stated that the CONUT score has good prognostic value in predicting the length of hospital stay in elderly patients, but it does not predict mortality^17^. Recent studies have found significant correlations between the severe CONUT score and the PNI of morbidity and mortality after coronary artery bypass graft surgery^18^.

In our study, we developed two models to assess the significance of the CONUT score. Model 1 incorporated variables such as age, history of HF, chronic kidney disease, hypertension, diabetes, and CAD. Model 2 included the CONUT score in addition to the variables in Model 1. We conducted a comparative analysis of the performance of both models using statistical methods and demonstrated that the CONUT score could serve as a substantial predictor of in-hospital mortality among CCU patients.

## CONCLUSION

Our study revealed that with thorough evaluation, the CONUT score could emerge as a significant determinant of in-hospital mortality among CCU patients.
